# An Integrated Genetic and Cytogenetic Map of the Cucumber Genome

**DOI:** 10.1371/journal.pone.0005795

**Published:** 2009-06-04

**Authors:** Yi Ren, Zhonghua Zhang, Jinhua Liu, Jack E. Staub, Yonghua Han, Zhouchao Cheng, Xuefeng Li, Jingyuan Lu, Han Miao, Houxiang Kang, Bingyan Xie, Xingfang Gu, Xiaowu Wang, Yongchen Du, Weiwei Jin, Sanwen Huang

**Affiliations:** 1 Institute of Vegetables and Flowers, Chinese Academy of Agricultural Sciences, Sino-Dutch Joint Lab of Horticultural Genomics, Opening Lab of Genetic Improvement of Agricultural Crops of Ministry of Agriculture, Beijing, China; 2 National Maize Improvement Center of China, Key Laboratory of Crop Genetic Improvement and Genome of Ministry of Agriculture, Beijing Key Laboratory of Crop Genetic Improvement, China Agricultural University, Beijing, China; 3 USDA, ARS, Vegetable Crops Research Unit, Department of Horticulture, University of Wisconsin, Madison, Wisconsin, United States of America; University of Umeå, Sweden

## Abstract

The Cucurbitaceae includes important crops such as cucumber, melon, watermelon, squash and pumpkin. However, few genetic and genomic resources are available for plant improvement. Some cucurbit species such as cucumber have a narrow genetic base, which impedes construction of saturated molecular linkage maps. We report herein the development of highly polymorphic simple sequence repeat (SSR) markers originated from whole genome shotgun sequencing and the subsequent construction of a high-density genetic linkage map. This map includes 995 SSRs in seven linkage groups which spans in total 573 cM, and defines ∼680 recombination breakpoints with an average of 0.58 cM between two markers. These linkage groups were then assigned to seven corresponding chromosomes using fluorescent *in situ* hybridization (FISH). FISH assays also revealed a chromosomal inversion between *Cucumis* subspecies [*C. sativus* var. *sativus* L. and var. *hardwickii* (R.) Alef], which resulted in marker clustering on the genetic map. A quarter of the mapped markers showed relatively high polymorphism levels among 11 inbred lines of cucumber. Among the 995 markers, 49%, 26% and 22% were conserved in melon, watermelon and pumpkin, respectively. This map will facilitate whole genome sequencing, positional cloning, and molecular breeding in cucumber, and enable the integration of knowledge of gene and trait in cucurbits.

## Introduction

The *Cucurbitaceae* family comprises about 120 genera and 800 species, including many economically important vegetable and fruit crops such as cucumber (*Cucumis sativus* L.), melon (*C. melo* L.), watermelon (*Citrullus lanatus* (Thunb.) Matsum. & Nakai), squash and pumpkin (*Cucurbita spp*.) [1]. Cucurbits are mostly prostrate or climbing herbaceous annuals that have coiled tendrils and they are characterized by having unisexual flowers and inferior ovaries. Although cucurbits vary in chromosome numbers, their genome sizes have not changed as significantly as in some other botanical families like Brassicaceae and Poaceae (e.g., cucumber: 2n = 2*x* = 14, 367 Mb; melon: 2n = 2*x* = 24, 480 Mb; watermelon: 2n = 2x = 22, 430 Mb, and squash and pumpkin: 2n = 2*x* = 40, 539 Mb) [2]. It seems that chromosome numbers of cucurbits correlate directly with their genome sizes. Differences in cucurbit genome size might be attributable to the structure and position of centromeres and telomeres, and the other repeat-related genomic elements. Genomic resources for cucurbits are scarce, and high density genetic linkage maps have not been reported for cucurbit species. This lack of genomic information seriously hampers genome assembly and genetic analysis in cucurbits.

In the genus of *Cucumis*, cucumber is the only species with a haploid chromosome number of seven (for other *Cucumis* species, basic number = 12). It is cross-incompatible with other *Cucumis* species and consequently, cucumber has a narrow genetic basis within domesticated market types [3]. India was thought to be the center of origin and domestication of this species where two botanical varieties *C. s.* var *sativus* L. (cultivated) and the feral form *C. s.* var *hardwickii* (R.) Alef coexist.

Unsaturated cucumber linkage maps have been developed using morphological traits, isozymes, and molecular markers, where markers (<300 loci) were often positioned in more than seven linkage groups [4]–[8]. Cytogenetic maps have also been constructed in cucumber using C-banding and fluorescence *in situ* hybridization (FISH) [9]–[11], allowing identification of seven morphologically distinct chromosomes. However, none of the maps ware integrated with cytogenetic map. Marker-trait associations have been effective in achieving selection gain for yield components during marker-assisted backcrossing [12], [13], but marker-assisted selection (MAS) of quantitative trait loci seems to be unpredictable due to lack of a high-resolution genetic map [14].

The availability of high-density maps in cucumber would facilitate whole genome sequencing and positional cloning, enhance MAS, and provide opportunities to investigate synteny among cucurbit species (e.g., cucumber and melon). SSRs (simple sequence repeats) or microsatellites are tandem repeats of short DNA sequences ranging in length from one to six base pair (bp), which are abundant and ubiquitous in all eukaryotic genomes [15], [16]. Because of their high level of polymorphism, ubiquity, and co-dominance, SSRs have become a valuable source of molecular markers in genetic analysis [17]. Only a limited number of SSR markers, however, have been developed for cucumber through exploiting EST sequences and screening genomic libraries [5], [18], [19], which has hampered use of molecular markers in genetic analysis and MAS in cucurbits.

We present herein the development of a saturated SSR-based cucumber linkage map employing 3× Sanger shotgun sequences. We used FISH to assign linkage groups to a cytogenetic map and to define chromosomal rearrangements between *C. sativus* var. *sativus* and var. *hardwickii*. The integrated genetic-cytogenetic map described herein provides a platform for genetic and genomic analysis that does not currently exist in cucurbits.

## Materials and Methods

### Plant and DNA materials

Two mapping populations were used for linkage mapping in this study. The first one consisted of 77 F_6_-F_8_ recombinant inbred lines (RILs) derived from the inter-subspecific cross between Gy14 and PI 183967 [6]. Gy14 is a North American processing market type cucumber cultivar and PI 183967 is an accession of *C.s.* var. *hardwickii* originated from India. Another population derived from an intra-subspecific cross (i.e., *C. s.* var *sativus* line 9930 × line 9110 Gt) consisted of 130 F_7_-F_8_ RILs, which was used for comparative analysis of marker clustering in the map of the inter-subspecific cross.

Eleven cucumber inbred lines were employed for genetic diversity studies with SSR markers. These 11 lines represented six market types worldwide: ‘Chinese Long’ type (228, 9930 and Xintaimici), Southern China type (Baiyesan and 00956), Southwestern China type (Xishuangbanna-1, Xishuangbanna-2), European greenhouse type (65 G and 9110 Gt), American slicing type (Marketmore 76), and Japanese type (185). All these inbred lines were from the Institute of Vegetables and Flowers, Chinese Academy of Agricultural Sciences.

Seeds for two melon inbred lines (*Cucumis melo* var. *saccherinus* cv.3A832 and *C. melo* var. *chinensis* cv. 4G21), two watermelon lines (*Citrullus lanatus* var. *lanatus* cv. 97103 and *C. lanatus* var. *citroides* PI 296341), and two squash lines (*Cucurbita moschata Duch* cv. bush#10 and *C. maxima* cv. Mengri) were kindly provided by Prof. Yong Xu and Dr. Jianshe Wang (Beijing Vegetable Research Centre, Beijing). These lines were used to test the cross-species transferability of SSR markers developed in cucumber.

Total DNA was isolated from expanding leaves of three-week old plants using the modified CTAB method [20].

### Development of SSR markers

**Figure 1:**
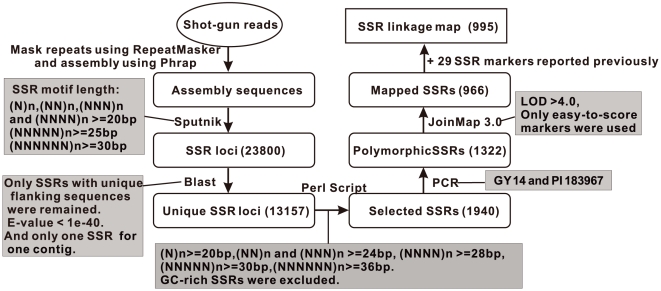
Flow chart of development of cucumber SSR markers.

The process of development of SSR markers employed is presented in Figure 1. Whole-genome “shotgun reads” with 3× cucumber genome coverage were masked for repetitive sequences using RepeatMasker (www.repeatmasker.org), and then were assembled using Phrap [21], resulting in large number of contig and singleton DNA sequences. Repeat motifs with length of at least 20 bp were identified from these sequences using a modified Sputnik program (modification specified in *C. abajian*, http://abajian.net/sputnik/). The repeat number threshold was designated as more than five, and each putative SSR locus was defined by a SSR motif and associated up and down stream flanking sequences of 200 bp. All putative loci were compared to contig and singleton sequences by BLAST analysis at an E-value cutoff of 1e-40. Putative locus with single hit was defined as a unique locus for use in map construction. If two or more unique loci were identified in one contig, only the one with the longest repeat motif was chosen for mapping to avoid redundant mapping effort. The minimum repeat number for selected MNRs (mono-nucleotide repeats), DNRs (di-nucleotide repeats), TNRs (tri-nucleotide repeats), TTRs (tetra-nucleotide repeats), PNRs (penta-nucleotide repeats) and HNRs (hexa-nucleotide repeats) was chosen to be 20, 12, 8, 7, 6 and 6, respectively.

Primer pairs were designed for these unique SSR loci using the Primer 3.0 program [22] with product sizes ranging from 130 to 220 bp. All primers were synthesized by Sangon Biological and Engineering Company, Shanghai, China.

### DNA amplification and electrophoresis

Each polymerase chain reactions (PCR) was performed in a 15 µl volume containing approximately 20 ng template DNA, 1×buffer, 0.5 unit *Taq* DNA polymerase (Tiangen Biological Company, Beijing, China), 20 ng of forward and reverse primers, 2 mM dNTPs. Optimized PCR thermocycling incorporated a denaturation step of 5 min at 94°, followed by 35 cycles of 15 sec at 94°, 15 sec at 55°, 30 sec 72°, and a final extension at 72° for 4 min. Subsequently, 3 µl of the PCR product was employed for electrophoresis in 6% polyacrylamide gel according to [23].

### Linkage map construction for cucumber

The markers with more than three missing genotype data were excluded, and the remaining marker data were used in linkage analysis with JoinMap program version 3.0 [24]. Initial linkage groups (LGs) were established at a LOD threshold of 10. The mapping data for those markers placed in these LGs were graphically displayed according to their orders in each LG using Microsoft Exel via a conditional cell formatting formula. In this display, the data points where the genotype data were in disagreement with both flanking data were defined as “singletons”. The markers with more than five “singletons” were excluded, and then the marker orders were reordered. This process was iterated until deleting markers did not affect the marker orders, and thus the framework genetic map was constructed. The deleted marker data were inspected visually again and corrected if there were errors. Based on the framework map, the markers which were not grouped into the above LGs were added by decreasing the LOD threshold step by step. The minimum LOD of 4 was used, and only the markers which did not affect the orders in the framework map were included. Also the reevaluated markers which had been excluded were added again, and the markers which did not affect the framework map were included in the final map. According to the final map, the data were again graphically displayed as described above. Co-segregating markers were manually determined using this display and defined as a “filled bin” [25]. A bin signature comprises the consensus segregation pattern of marker loci, which does not recombine and was thus incorporated in the bin. If adjacent “filled bins” differed by two or more genotype data that indicating two or more recombinations between them, the matching “empty bins” were included. The filled and empty bins were numbered consecutively, and thus resulting in a skeleton bin map. The consensus genotype data for filled bins were then used to calculate genetic distances. Information of the mapped SSR markers is listed in Table S1.

### Efficacy of SSR markers for genetic diversity analyses

The efficacy of each SSR marker for germplasm discrimination among 11 genetically diverse cucumber inbred lines (see Plant and DNA materials) was estimated with polymorphism information content (PIC) using the formula [26]:
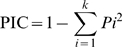
, where *k* is the total number of alleles detected for a SSR marker and *Pi* is the frequency of the *i*th allele.

The heterozygosity for each marker based on the genotype data with the RIL mapping population was calculated with POPGEN32 software (www.ualberta.ca/~fyeh/).

### Florescent *in situ* hybridization analysis

Type I/II, III, IV and rDNA (45S) cucumber DNA repeats [10] and fosmid clones used for fluorescent *in situ* hybridization (FISH) analysis were provided by Beijing Genomics Institute, Beijing, China. The fosmid library was constructed from inbred line 9930 which was also used for whole genome sequencing. Selected fosmid clones were end-sequenced. Fosmid clone end sequences were then placed in the assembled contigs of the 3× shotgun sequences and used to screen the fosmid library to identify clones carrying genetically defined SSR sequences (Tables S2). Selected fosmid clones were used as probes in FISH analysis. Chromosome preparation and FISH procedures followed Jiang et al [27]. Briefly, root tips were harvested from germinated seeds, pretreated in 4°C water for 2–4 h to capture pro-metaphase and metaphase cells, and fixed in Carnoy's solution (3 ethanol: 1 glacial acetic acid). Root tips were then macerated in 2% cellulose and 1% pectolyase at 37°C for 2 h, and squashes were prepared using the same fixative.

DNA probes were labeled with either biotin-dUTP or digoxigenin-dUTP (Roche, Indianapolis, IN, USA) via nick translation and detected with anti-digoxigenin antibody coupled with Rhodamin (Roche) or anti-avidin antibody conjugated with FITC (Vector Laboratories), respectively. Chromosomes were then counterstained using 4, 6-diamidino-2-phenylindole (DAPI) in an antifade solution Vectashield (Vector Laboratories, Burlingame, CA), and images were captured digitally using a Sensys CCD (charge coupled device) camera (QIMAGING, RETIGA-SRV, FAST 1394) attached to an Olympus BX61 epifluorescence microscope using Image-Pro Plus 6.0 software (Media Cybernetics) to capture grey scale images that were adjusted with Adobe Photoshop 6.0 software.

## Results

### High Density Genetic Map

#### Marker development

A total of 23,800 putative SSR sequences were identified from whole-genome 3× shot-gun sequencing. The frequencies of different types of SSRs in this genome were listed in Table S3. To reduce the nonspecific amplification, SSR primers with multiple homologues in the assembly were excluded (Figure 1). If a sequence contains more than one SSR, only the one with the longest motif was chosen to reduce marker clustering. Thus, the number of putatively unique SSRs was reduced to 13,157, from which 1,940 with the longest repeat motifs were selected for polymorphism screening between RIL mapping population parental lines (Gy 14 and PI 183967). Of the 1,940 SSRs, 1,322 (68.1%) were polymorphic between Gy 14 and PI 183967 (Figure 1). The polymorphism level between Gy14 and PI 183967 was also tested with 200 previously reported SSRs [5], [19], [28], of which 167 could be amplified in the two lines, and of which only 40 (24%) were polymorphic

#### Map overview

**Figure 2:**
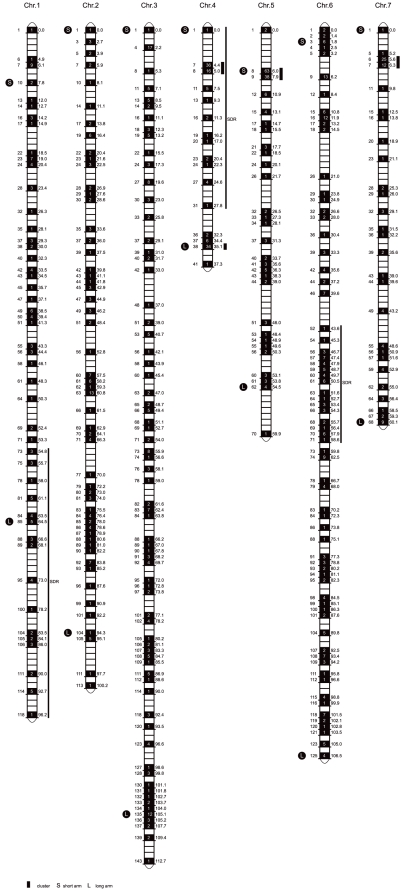
Cucumber linkage map developed from the present study. The Bin names and genetic distances in cM are respectively listed on the left and right of the chromosomes. The number of SSR markers in each filled Bin is indicated in the boxes. White boxes indicated a recombination event with no markers. The short and long arms are indicated with S and L, respectively SDR = segregation distortion region.

Nine hundred and ninety five SSR loci were mapped in seven linkage groups spanning 572.9 cM. In total, 678 recombination events (bins) were identified, where 311 (46%) Bins were filled by one or more markers (Table 1, Fig 2). Since the cucumber genome size is approximately 367 Mbp [2], the map defined herein represents average genetic and physical intervals of ∼0.6 cM and ∼370 Kb per marker, respectively, making it the most saturated linkage map in the Cucurbitaceae to date.

**Table 1:** Summary of the cucumber genetic map with RIL mapping population from the inter-subspecific cross between Gy14 and PI 183967.

Chr.	No. Markers	cM	Density (cM/marker)	Recombination events	Filled bins	Pachytene chr. size^a^ (µm)	Euchromatic chr. size^b^ (µm)
1	118	96.2	0.82	118	47	107	91
2	126	100.2	0.80	113	54	103	81
3	187	112.7	0.60	143	70	129	121
4	114	37.3	0.33	41	16	102	88
5	160	59.9	0.37	70	29	95	85
6	203	106.5	0.52	125	68	110	103
7	87	60.1	0.69	68	27	73	64
Total	995	572.9	0.58	678	311	719	633

a Pachytene chromosome length was based on [9].

b Euchromatic chromosome length was deduced from Koo et al (2005).

#### Marker distribution

The correlation is high between pachytene length and the number of markers per chromosome (*r* = 0.71) and between euchromatin length vs. the number of total markers per chromosome (*r* = 0.82) (derived from Table 1). Thus, the majority of mapped markers were obtained from the euchromatic, non-repetitive regions. Cytogenetic analysis in the present study also supported this conclusion. FISHing of interphase or pachytene chromosomes revealed that most fosmid clones anchored by the markers were located in the fainter DAPI staining regions or the euchromatin regions (Data not shown). No obvious centromeric clustering of mapped markers was observed (Figure 2).

#### Recombination suppression

Due to marker clustering (≥20 markers per two adjacent bins), the mapping distance (cM) or genetic recombination (Bins) on chromosomes 4, 5, and 7 was dramatically less than that detected on other four chromosomes (Table 1, Figure 2). Moreover, while chromosomes 5 and 7 each had one cluster, chromosome 4 had two clusters and hence the shortest mapping distance (37.3 cM) and least number of recombination events (41 Bins). Taken collectively, these four clusters included 225 (22.5%) of all 995 mapped loci.

Chromosomal rearrangement is known to cause recombination suppression [29], which may be the cause of the marker clustering observed in the present study. Each marker cluster possessed both parental alleles, and thus were not hemizygous. These “suppression regions” were examined by comparative recombination analysis between the inter-subspecific map (Gy 14× PI 183967) and an intra-subspecific map ( *C. s.* var *sativus* line 9930× line 9110 Gt). For example, the cluster on chromosome 5 of the inter-subspecific map spanned 1.9 cM, but it was increased dramatically to 61 cM in the intra-subspecific map. Similarly, the cluster on chromosome 7 was also notably different between the intra-subspecific (18 cM) and the inter-subspecific map (0.9 cM), suggesting possible structural changes between chromosomes of Gy14 and PI 183967 that resulted in suppression of meiotic recombination. Indeed, one reversion was identified in our molecular cytogenetic analysis as described below.

#### Segregation distortion regions

Three segregation distortion regions (SDRs) were detected on chromosomes 1, 4 and 6 (Figure 2). The SDR on the long arm of chromosome 1 was relatively large spanning from Bin 73 to Bin 118 including 48 SSR marker loci. The segregation of ∼62% (71/114) of SSRs mapped to chromosome 4 was distorted forming an SDR that spanned the entire short arm and the proximal portion of the long arm. The SDR on chromosome 6 spanned from Bins 52 to 71 of the long arm. All SSR marker loci within these SDRs were associated with the *C. sativus* var. *hardwickii* parent (PI 183967), indicating that possibly interacting allele pairs with strong effects on pollen or embryo viability or germination are located on the SDRs and that the ‘wild’ alleles confer stronger viability than the ‘domesticated’ ones.

### Cytogenetic Characterization

#### Assigning linkage groups to chromosomes

**Figure 3:**
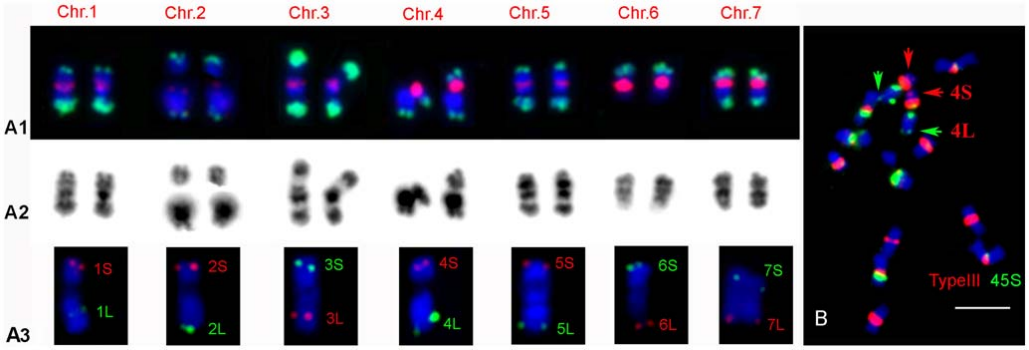
Integration of the seven linkage groups of cucumber with individual chromosomes. (A1) Distribution of Type I/II (green) and Type III (red) repeats on cucumber chromosomes. (A2) DAPI staining was converted to black and white images. (A3) Localization of chromosome-specific fosmid clones on both arms of individual chromosomes, genetic location of arm-specific fosmid clones are indicated in Figure 2. (B) Localization of fosmids 4S (red) and 4L (green) together with Type III (red) and 45S rDNA (green) repeats on the mitotic chromosomes. Bar = 2.5 µm.

FISH analysis was used here to establish the relationships between linkage groups and chromosomes in cucumber. Three to five SSR markers located in the distal ends of each linkage group were selected to screen a fosmid library developed from inbred line 9930. To identify individual chromosome, these fosmid clones were FISH-mapped on mitotic chromosomes (Figure 3A), which were then reprobed with two tandem repeat sequences (Type III and 45S) (Figure 3B) whose distribution patterns on each haploid chromosome are known [10]. Using this strategy, each fosmid clone defining a single locus was assigned to a chromosome allowing integration of all seven linkage groups into chromosomes (Figure 3A). The short arm/long arm orientation of each linkage group could be established based on the positions of fosmid FISH analysis. i.e., physical locations of the chromosome-specific fosmid clones represent chromosome locations of corresponding SSR markers used during fosmid clone screening (Figure 2 and Figure 3). Furthermore, the 14 chromosome arm-specific fosmid clones can also serve as convenient and reliable cytological markers in the future cytogenetical studies of cucumber.

#### Inter-subspecific chromosomal variation

**Figure 4:**
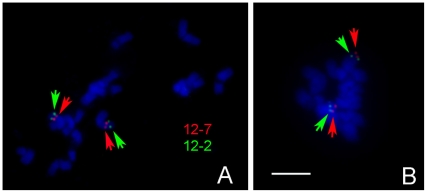
An inversion detected by FISH on cucumber chromosome 5 between GY 14 and PI183967. (A) Locations of the fosmid 12-7 (SSR20648, red) and 12-2 (SSR13340, green) on chromosome 5 of GY14. Red signals are located closer to the telomere than green signals. (B) Locations of the fosmid clones on chromosome 5 of PI 183967. Note that green signals are closer to the telomere. Bars = 3 µm.

The discrepancy between the inter-subspecies map and the intra-subspecies map on recombination suppression strongly suggested chromosomal variations between the two subspecies. To test this hypothesis, the marker cluster on chromosome 5 containing 101 markers (Figure 2. Bin8–9) were analyzed in detail. Two SSR markers (SSR13340 and SSR20648) from this cluster were chosen to screen fosmid clones. Next two positive clones were used in FISH-mapping to assign each clone to a specific region of chromosome 5. It was found that the order of the two clones on the two chromosomes of two subspecies was the opposite, indicating presence of a chromosomal inversion that may be the reason of recombination suppression in this species (Figure 4).

## Discussion

In this study, we described the development of highly polymorphic SSR markers using whole genome shotgun sequences leading to the construction of the first high-density genetic map in cucumber. This linkage map was then used as a reference in FISH analysis to define the first integrated genetic-cytogenetic map in this species. The integrated map will be useful in facilitating whole genome assembly, molecular breeding, and positional gene cloning in cucumber, and could act as a reference map for comparative analysis in other cucurbit genomes.

### Highly polymorphic markers for genetic analysis of cucurbits

According to the formulae of Lander and Waterman [30], the 3× shotgun sequencing applied herein covered about 95% of the cucumber genome and provided an opportunity to “mine” highly polymorphic markers in cucumber that has a narrow genetic base. It seems that the degree of polymorphism of these SSR markers is positively related to the length of repeat motifs [31], [32]. The whole-genome scan initially identified nearly 23,800 putative SSRs of which 1,940 with the longest motifs were selected for polymorphism screening and map construction. The average repeat motif length for the selected SSRs was 39.4 bp, which was longer than that of previously reported SSRs (27.6 bp). The polymorphism level between Gy14 and PI 183967 in these new SSRs was almost three times as high as those reported previously. This result demonstrated the power of whole genome sequences in enhancing genetic analysis of an under-investigated crop with a narrow genetic base. In turn, the saturated genetic map will help anchor DNA sequence assemblies onto chromosomes to generate a map-based genome sequence of cucumber.

### Saturation of the euchromatic region of the cucumber genome

Since the SSR markers developed herein were created from non-repetitive regions of the genome, it might be hypothesized that these markers were associated predominantly with the euchromatic or gene-rich regions of the genome. Three lines of evidence supported this hypothesis. First, the number of mapped loci per chromosome was positively correlated with the euchromatic pachytene length of each chromosome (Table 1). Second, marker clustering around the centromeric region was not detected (Figure 2). Last, the 12 FISH-mapped fosmid clones tagged with 12 SSR markers were evenly distributed across the length of chromosome 6 (data not shown). Only one SSR marker mapped to the heterochromatic region of the chromosome.

### Chromosomal variation in cucumber

The mapping population used in this study was derived from a cross between two distinct botanical varieties [6]. PI 183967 belongs to *C sativus* var. *hardwickii* which is a native to the Sub-Himalayan region of India and is believed to be a wild feral form of cucumber [33]. The FISH analysis with SSR markers from one cluster revealed what is most likely a paracentric inversion on chromosome 5 (Figure 4), which may well explain the high degree of suppression of genetic recombination in this region using the Gy14× PI183967 RIL mapping population. This observation also supports the notion by Chung et al. [34], that *var. hardwickii* is a feral form of *var. sativus*. Although these botanical varieties are cross-compatible and var. *hardwickii* possesses several economically important traits (e.g., multiple and sequential fruiting habit [35]; downy mildew resistance [36]; root-knot nematode resistance [37]; cucumber mosaic virus [38]), their introgression into elite commercial germplasm has been difficult. If genes controlling such traits are located in the regions with chromosomal variation, then fixation of positive alleles in commercial types would likely be complicated.

### Use of SSRs in cucurbit breeding

**Figure 5:**
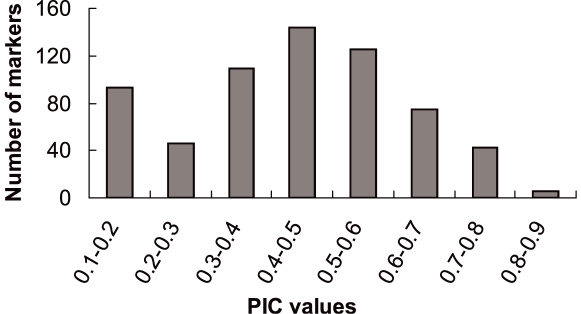
Distribution of PIC values for 642 SSR markers in cucumber. PIC values were calculated from PCR among 11 cucumber inbred lines.

The narrow genetic base of cucumber has so far limited wide use of marker technology in crop improvement [39]. The SSR markers developed from the present study were used to examine the genetic affinity of diverse cucumber inbred lines and evaluate their potential in marker-assisted selection. Approximately 65% of the 995 SSRs examined were polymorphic in these 11 lines. The PIC values ranged from 0.17 to 0.84, with an average value of 0.44 indicating that the SSR markers employed provided for a robust discrimination among this germplasm array. Moreover, the SSRs with PIC values from 0.4 to 0.6 were most common, and ∼250 SSRs with PIC value >0.5 were highly polymorphic (Figure 5). On the other hand, based on the genotype data of these markers with the RIL mapping population the average heterozygosity was calculated to be 0.48 and approximate to the PIC value derived from the 11 lines. Therefore, these highly informative SSR markers would likely be useful in tracing economically important traits in breeding populations.

The map constructed herein may have a broader scope of deployment and use in cucurbit breeding. An appreciable number of the SSR markers were able to amplify products in melon [487 (48.9%)], watermelon [258 (25.9%)] and pumpkin [221 (22.2%)] (Table 2). Moreover, these SSR markers detected relatively high levels of polymorphism in these species (melon, 39.6%, watermelon, 46.5%, and pumpkin, 54.8%). Thus, these markers are also potential useful in these crop species.

**Table 2:** Cross-species transferability of 995 cucumber genomic SSR markers in melon, watermelon and pumpkin.

	Melon	Watermelon	Pumpkin
Amplified loci	487 (48.9%)	258 (25.9%)	221 (22.2%)
Polymorphic loci	193 (19.4%)	120 (12.1%)	121 (12.2%)
Polymorphism (%)	39.6	46.5	54.8

The percentages of amplified and polymorphic loci were calculated based on 995 SSR markers.

## Supporting Information

Table S1 (0.25 MB XLS)

Table S2 (0.04 MB DOC)

Table S3 (0.03 MB XLS)
