# Supplementary material for: An Integrated Genetic and Cytogenetic Map of the Cucumber Genome
Source: PLoS One. 2009 Jun 4;4(6):e5795. doi: 10.1371/journal.pone.0005795 (PMC2685989; doi:10.1371/journal.pone.0005795)
Supplement: Table S2 — (0.04 MB DOC) [file pone.0005795.s002.doc]

**Table S2. Summary of the fosmid clones selected for integration of the chromosome map and its genetic map in cucumber.** Listed are SSR markers used to search fosmid, their genetic location and physical location (Figure 3).

| Chr. No. | SSR markers | Gentic locations (cM) | Selected Fosmids | Physical locations |
| --- | --- | --- | --- | --- |
| 1 | SSR00190 | 7.8 | fbd0-0737-D07 | 1S |
| 1 | SSR20354 | 64.5 | fbe0-0177-D10 | 1L |
| 2 | SSR00184 | 0 | fbd0-0022-B06 | 2S |
| 2 | SSR30665 | 94.3 | fbe0-0304-A09 | 2L |
| 3 | SSR03049 | 0 | fbe0-0472-B12 | 3S |
| 3 | SSR23517 | 105.1 | fbe0-0001-D11 | 3L |
| 4 | SSR03598 | 0 | fbd0-0280-E09 | 4S |
| 4 | SSR21197 | 34.7 | fbd0-0802-E07 | 4L |
| 5 | SSR18593 | 6.0 | fbd0-0670-B01 | 5S |
| 5 | SSR12502 | 54.5 | fbd0-0184-A05 | 5L |
| 6 | SSR00158 | 1.8 | fbe0-0620-F09 | 6S |
| 6 | SSR06271 | 106.5 | fbd0-0131-G09 | 6L |
| 7 | SSR15477 | 0 | fbe0-0624-A03 | 7S |
| 7 | SSR17062 | 88.9 | fbd0-0556-D09 | 7L |
